# Thalamocortical network interruption: A fresh view for migraine symptoms

**DOI:** 10.3906/sag-2005-21

**Published:** 2020-11-03

**Authors:** Hayrunnisa BOLAY

**Affiliations:** 1 Department of Neurology and Algology Faculty of Medicine, Gazi University, Ankara Turkey

**Keywords:** Migraine, headache, sensory discrimination, thalamocortical network, sensory integration, CSD

## Abstract

Migraine is a multifaceted brain disorder where multisensory disturbances are associated with headache. Yet sensory symptoms are conventionally justified by dysfunctions confined to the cerebral cortex, a perspective through the complex interplay of thalamocortical network would provide the entire picture, more pertinent to the central sensory processing. It is important to consider thalamus as a hub that integrates multiple domains via extensive connections among anatomically and functionally separate cortical areas. Accordingly, cortical spreading depression (CSD), implicated in migraine pathophysiology can be seen as a tool to disconnect thalamocortical network by functionally eliminating cerebral cortex. Hence, including thalamic reticular nucleus and higher order thalamic nuclei, which conveys the information transthalamically among visual, somatosensory, language and motor cortical areas, would greatly improve our current understanding of migraine.

## 1. Introduction

Migraine is the most prevalent brain disorder under age of 50 all around the globe and the first cause of disability due to severe headache and associated sensory disorders [1]. The characteristic features of pain are one sided, severe, pulsating headache attacks, worsening by daily movements and lasting 4–72 h. Accompanying sensorial symptoms suggesting hyperresponsivity in the somatosensory, visual, auditory, and olfactory systems are distinguishing features of migraine attacks and multisensory stimuli may worsen the headache severity [2]. Additionally, prior to the headache phase more pronounced sensorial disruptions can occur in the visual and somatosensory system such as scintillations, scotoma or paresthesia, numbness in the extremities. These slowly propagating sensory deficits are recognized as aura symptoms and implicated by underlying cortical spreading depression (CSD) waves [3]. 

## 2. CSD as a tool disconnecting the cortex from thalamus

CSD is a neuronal and glial depolarization wave that slowly propagates within the cerebral cortex, causing transient electrical and functional silence of the involved area. Propagation of massive depolarizations and accompanying electrical DC shift and regional blood flow increase along the cortex, left long-lasting decreased neuronal excitability and reduced regional blood flow behind [3, 4]. The sensory aura symptoms are considered solely due to cerebral cortical dysfunction by CSD. However, this conventional approach disregards several facts that i) propagation of CSD in gyrencephalic brains such as humans, primates and even felines is very restricted [4], ii) cerebral cortex functions in a synchrony with thalamus and brain network systems. In that sense CSD was essentially used to generate functional ablation of cerebral cortex in thalamic studies [4]. 

## 3. Hub function of higher order thalamic nuclei

Understanding the function of cerebral cortex requires knowledge of complex interaction between the cortex and the thalamus, known as thalamocortical network. The cerebral cortex and the thalamus operate in continuous interaction during the process of any information particularly related to somatosensory, visual and auditory sensory systems, movement, language, cognition and consciousness [5,6]. The thalamus sends projections to majority of the cerebral cortex, and receives cortical outputs in return. Thalamic nuclei transmitting peripheral sensory inputs to the primary cerebral cortical areas are considered as the first-order relay nuclei such as the ventral posteromedial (VPM) and lateral geniculate nucleus (LGN) that relay cephalic somatosensory inputs and visual stimuli respectively. Likewise, the higher-order thalamic nuclei receive inputs from the deep cerebral cortical layers and relay information from one cortical area to another (Figure). By this means, higher-order thalamic nuclei function as hubs that synchronize distant cerebral cortical areas via transthalamic cortico-cortical connections [7]. 

**Figure:**
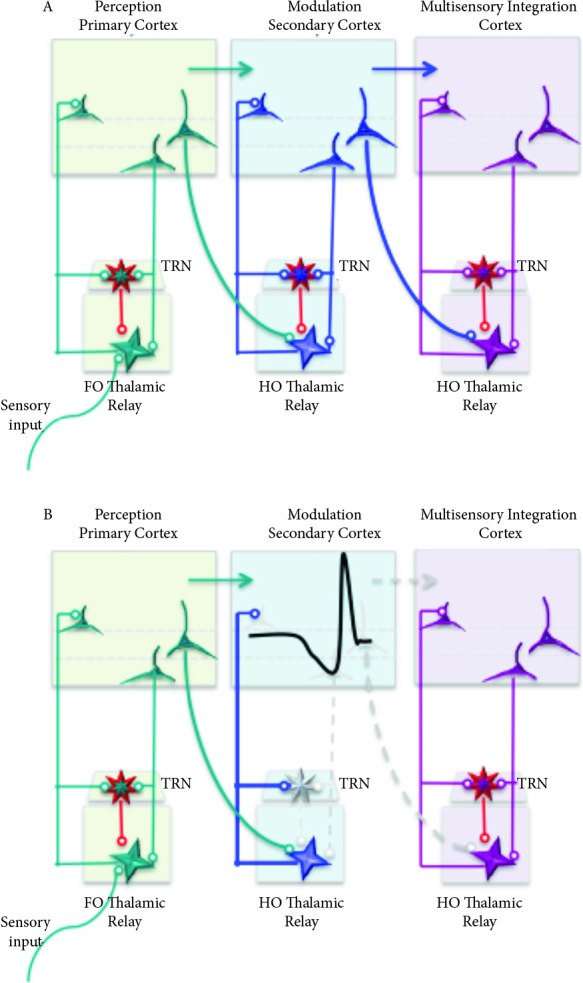
Schematic diagram of the complex thalamocortical network properties and the possible consequences of CSD. A) Thalamus transmits external sensory input to the primary sensory cortex through first-order thalamic relay nuclei. Cortical information is transferred via direct cortico-cortical pathways, and also transthalamically through higher order thalamic relay nuclei to different cortical regions. Hence the thalamus can be considered as an integrative hub for higher multisensory and sensorimotor brain networks. B) CSD induces loss of spontaneous and synaptically driven neuronal activity locally in the cerebral cortex. Absence of such a powerful drive from cortical neurons within this complex network would lead to altered firing properties of thalamic neurons that change the impulse trafficking through thalamus to other cortical areas within the hub. Abrupt cessation of cortical excitatory glutamatergic drive from cortex, leads to reduced GABAergic output from TRN on to thalamic relay nuclei and increased sensory relay to the cortex. Additionally, propagation of SD waves into TRN would eliminate GABAergic inhibition on thalamic relay neurons, leading to enhanced sensory transmission to the cerebral cortex. Subsequent sensory disturbances as aresult of thalamocortical network interruption may include i) increased response to sensory stimulus in multiple domains such as photophobia, phonophobia, allodynia, ii) multisensory and sensorimotor integration problems such as impaired SAI[16], iii) sensory discrimination impairment such as STD prolongation [12–14], iv) complex aura symptoms manifested by concomitant visual, somatosensory, language dysfunctions [4], and v) impaired cognitive modulation of sensory stimuli.

## 4. Strategic role of thalamic reticular nucleus (TRN)

The deep cortical cells particularly from the 6th layer, also project to inhibitory neurons in the thalamic reticular nucleus (TRN) and modulate the frequency-dependent thalamic function [6–8]. The TRN consists of GABAergic inhibitory neurons and surrounds the dorsal thalamus. While the TRN exclusively projects to the thalamus and inhibits thalamic drive to the cortex, its neurons are driven by the first- and higher-order thalamic nuclei as well as the cerebral cortex [5,6]. TRN involvement during CSD was demonstrated in awake, consciously behaving experimental animals [9,10]. CSD induced SD waves were recorded in TRN and neuronal activation was demonstrated. It is likely that either SD in the cerebral cortex could provoke SD in TRN or SD waves propagate from cerebral cortex to the TRN, as subcortical structures are more susceptible compared to highly convoluted cerebral cortex in humans [4]. 

Involvement of the TRN may play a role in reduced sensory discrimination, lateral inhibition and increased response to sensory stimuli in different modalities such as light, sound and touch during migraine attacks. A subset of cortical deep projection neurons was shown to project selectively to higher order thalamic nuclei in mice and recently the higher order thalamic dysfunction was proposed to be involved in the development of migraine symptoms [4, 8]. 

## 5. Thalamocortical perspective to justify sensory symptoms

Instead of attributing sensory dysfunctions merely to the cerebral cortex, alternative view proposes a complex interaction of first- and higher-order thalamocortical areas along with TRN (Figure). A minimum critical volume of 1 mm3 rodent cortical brain tissue was estimated to be depolarized to ignite CSD [11]. Even if the propagation of CSD is blocked, such a depolarized cortical focus is sufficient to create a change in ongoing cortico-thalamic drive on thalamic relay nuclei and thalamic reticular nucleus. During CSD, abrupt cessation of cortical neuronal firing, would result in decreased excitatory glutamatergic drive from cortexto the thalamus, leading to a reduced GABAergic output from TRN on to thalamic relay nuclei and increased sensory impulses through thalamic relay to the cortex (Figure). That would account for the increased sensory excitation, sensory hyperresponsivity, reduced discrimination and reduced lateral inhibition and bidirectional role of sleep in migraine. Therefore, the processing of somatosensory stimulus and sensory discrimination was investigated in migraine patients [12–16]. For that purpose, somatosensory temporal discrimination (STD) test was employed. STD detects the temporal threshold to perceive two consecutive somesthetic stimuli as undoubtedly distinct [12–14]. STD was remarkably prolonged during the migraine attack while STD threshold values outside the migraine attacks were comparable to healthy volunteers [12]. STD studies also revealed that such a prolongation was specific to migraine attacks and was not detected during tension type headache, which is another common headache disorder [14]. It is an intriguing finding that discrimination of somatosensory stimuli applied to the hand that is transmitted through a pathway via thalamic ventral posterolateral nucleus (VPL), is disturbed during migraine attacks. Indeed, migraine headache is transmitted through a different pathway via trigeminal nerve to the thalamic ventral posteromedial nucleus (VPM). Hence, it is unlikely that prolonged temporal discrimination of somatosensory stimulus applied to hand is merely mediated as a direct result of trigeminal nerve or thalamic VPM activation. 

## 6. Interpretation of sensory dysfunctions in migraine 

Transient impairment in the ability of discriminating the exact entry of somatosensory stimuli suggested a central processing dysfunction. Further study was conducted by short afferent inhibition (SAI) paradigm using transcranial magnetic stimulation [16]. SAI evaluates integration of somatosensory input through the thalamic relay neurons in the sensorimotor cortex. The SAI results revealed that impaired sensorimotor integrity and reduced cortico-cortical inhibition between somatosensory and motor cortices were associated with migraine attacks [16]. SAI impairment during migraine attacks, indicated a disinhibition in the sensorimotor integration. Remarkably, the facilitation of motor responses to a conditioned sensory stimulus was detected even several hours prior to the onset of migraine headache [16]. Therefore, the possible involvement of higher order centers seems undisputable. 

In the higher order thalamic nuclei such as pulvinar, that would be manifested by the disrupted visual information processing and may be related to positive visual hallucinations superimposed on to a first-order incoming information, similar to the patients’ visual auras [4]. Also, higher order visual areas and somatosensory areas are associated with medial and anterior part of the pulvinar that is a multimodal sensory integration area [17]. Therefore, loss of any incoming information by CSD from one of the interconnected cortical areas such sensorimotor cortex or visual cortex to the pulvinar would change impulse trafficking and may yield multisensory sensory symptoms synchronously and/or sequentially.

## 7. Conclusion 

Thus, the view of thalamocortical network dysfunction would clarify positive aura symptoms and simultaneous manifestation of different symptoms related to distant cortical areas such as visual, sensorimotor and language cortices interconnected through a thalamic hub. Additionally, sensorial disruptions in more than one domain accompanying migraine headache can be attributed to multisensory integration dysfunction of the higher order thalamocortical network. Involvement of TRN would contribute sensory hypersensitivity in multiple sensory modalities, lateral inhibition and sensory discrimination problems associated with migraine headache. Additionally, activation of trigeminal pain nucleus through central pathways as a result of thalamocortical dysfunction could also contribute to the development of lateralized migraine headache.
